# Fertility Sparing Treatment in Young Patients with Early Endometrial Adenocarcinoma: Case Series

**DOI:** 10.12669/pjms.292.3280

**Published:** 2013-04

**Authors:** Mehri Jafari Shobeiri, Parvin Mostafa Gharabaghi, Heidarali Esmaeili, Elaheh Ouladsahebmadarek, Mahzad Mehrzad-Sadagiani

**Affiliations:** 1 Mehri Jafari Shobeiri, Associate Professor, Department of Gynecologic Oncology, Women's Reproductive Health Research Center, Tabriz University of Medical Sciences, Tabriz, Iran.; 2 Parvin Mostafa Gharabaghi, Associate Professor, Department of Gynecologic Oncology, Women's Reproductive Health Research Center, Tabriz University of Medical Sciences, Tabriz, Iran.; 3 Heidarali Esmaeili, Associate Professor, Department of Pathology, Tabriz University of Medical Sciences, Tabriz, Iran.; 4 Elaheh Ouladsahebmadarek, Associate Professor, Department of Gynecologic Oncology, Women's Reproductive Health Research Center, Tabriz University of Medical Sciences, Tabriz, Iran.; 5 Mahzad Mehrzad-Sadagiani, Associate Professor, Department of Infertility and Reproductive, Tabriz University of Medical Sciences, Tabriz, Iran.

**Keywords:** Endometrial carcinoma, Progestins, conservative treatment, Fertility preservation

## Abstract

***Objective:*** The aim of this study was to evaluate fertility-sparing therapy in young patients with endometrial carcinoma.

***Methodology:*** This prospective study was carried out on 8 patients with clinical and radio-graphic stage IA, well differentiated endometrioid adenocarcinoma of the endometrium in Alzahra hospital, Tabriz, Iran. Treatment comprised high-dose megestrol acetate. Dilatation and curettage was repeated every three months.

***Results:*** The mean age of the patients was 30 (SD,3.21) years (range 24-35). Of the 8 patients, 7 (87.5%) achieved complete response. The mean time to response was 6.5 months (range 3-9). Of the complete responders, 3 of 7(42.8%) had recurrence; one patient underwent immediate hysterectomy, and 2 were successfully treated with second-line therapy and both subsequently conceived. Conception occurred in 3 of 7 patients (42.8%), in two more than once, However successful pregnancy occurred only in two patients. One patient developed Concomitant ovarian adenocarcinoma.

***Conclusions:*** High dose progestin therapy can be an effective fertility-sparing treatment in young patients with well differentiated stage IA endometrial endometrioid cancer confined to endometrium. However, close follow up is required because of risks of conservative treatment.

## INTRODUCTION

Endometrial cancer is the most common malignant tumor of female genital tract in the world. Most patients are between the ages of 50 and 59 years. Up to 20-25% of uterine adenocarcinoma are diagnosed before the menopause, and approximately 5% before the age of 40 years.^1^^,^^2^

The treatment generally recommended for patients diagnosed with endometrial carcinoma is hysterectomy and bilateral salpingoophorectomy with or without lymphadenectomy which may be unacceptable to young women desiring further fertility. However in young patients conservative treatment with progestogens has been attempted, and the promising results has been reported.^3^^-^^5^

Endometrial endometrioid adenocarcinoma in patients under the 40 years is likely to be well differentiated. As highly differentiated tumors tend to retain their estrogen and progestron receptors and because progestins have an antiestrogenic effect on the endometrium, their use has been evaluated as a primary treatment for early clinical stage endometrial endometrioid cancer.^6^^-^^8^

For further perspective, we evaluated the outcome of a cohort of young women with endometrioid endometrial adenocarcinoma with clinical International Federation of Gynecology and Obstetrics (FIGO) stage IA, Garde 1 confined to endometrium by magnetic resonance imaging (MRI) who were treated by megestrol acetate as a fertility – sparing treatment.

The aims of this study were: 1. To find out the effect of treatment on their disease. 2. To find out the outcome of conservative management including primary, secondary and complete response, time to response, time to recurrence, recurrence rate, successful pregnancy rate (take home baby rate) and associated cancer.

## METHODOLOGY

All young patients with endometrioid endometrial adenocarcinoma clinical FIGO stage IA, well differentiated confined to endometrium by MRI were enrolled into the conservative protocol treatment using hormone therapy at the department Gynecologic Oncology, Alzahra teaching hospital, Tabriz, Iran, between 2002 and 2011. 

Inclusion criteria was age ≤ 35years, nulliparous, endometrioid adenococarcinoma, Grade 1 differentiation, no myometrial invasion being identifiable on MRI, no extrauterine spread by vaginal ultrasound and Computed Tomography Scan (CT SCAN), normal serum levels of CA125 (< 35 Iu/ml), carcinoembryonic antigen (CEA; <5 ng/ml), progesterone receptor positive (p_g_ R; by immunohistochemistry) and strong desire to preserve fertility. Excluded were patients with histopathology results of adenosquamous, clear cell, or papillary serous carcinoma.

Age at diagnosis, previous diagnosis of infertility and polycystic ovary syndrome, type of abnormal uterine bleeding (Menorrhagia, menometrorrhagia, or oligomenorrhea) were in dividually recorded as the characteristic features.

The initial diagnosis was based on the results of an outpatient endometrial biopsy. Dilatation and curettage (D&C) was performed in all patients before treatment, to collect specimens for repeating histologic examination, and also for estrogen receptors (ERs) and p_g_ R s by immunohistochemistry. All histology was reported by two pathologists experienced in the endometrial cancer.

All patients were counseled for that this form of hormone therapy is not standard treatment, and therefore requires serial D&C during follow up period. After informed consent was obtained, Megestrol acetate (Megace; Bristol- Myers Squibb, Princetone, NJ) 320mg/day for three months was commenced. Ethical approval was obtained from the Research Vice Chancellor Office, Tabriz University of Medical Sciences.

The first response assessment by D&C with general anesthesia was performed after three months' protocol treatment. Primary response was defined as benign endometrial histopathalogy on D&C specimen. The patient with no response was given the option of definitive surgery or to continue the same protocol treatment for three more months. Secondary response was defined as the benign histopathologic result after three extra months of treatment. If patient did not respond to the second course of treatment, definitive surgery or second- line regimen was recommended. In the second–line regimen, a GnRHa, triptorelin acetate (Decapeptyl CR; Ferring Pharmaceuticals, GmbH) 3.75- mg intramuscular injection monthly, was added to the same megace protocol treatment for three months.

**Fig.1:**
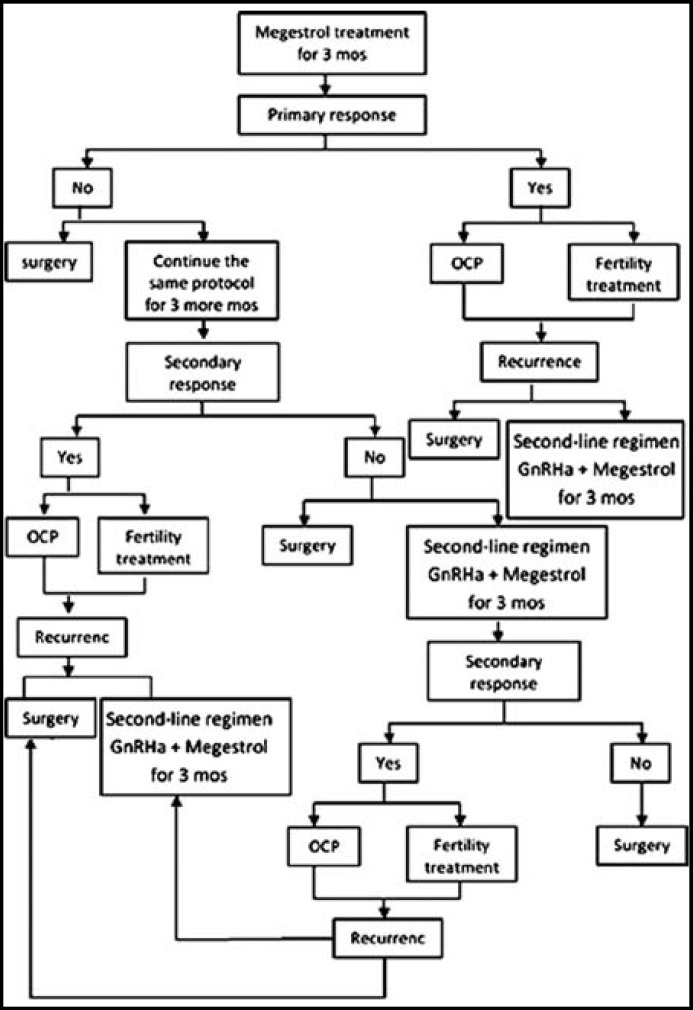
Summary of the methodology

After primary and secondary response, the patients were closely followed in the clinic by the history, serial serum CA125 and CEA, vaginal examination, vaginal ultrasonography every two months, and MRI every six months. D&C was performed every three months, and whenever there were suspicious signs or symptoms or abnormal findings on imaging studies. Those who urgently needed to conceive were referred to the infertility clinic to start fertility treatment, whereas the others were given oral contraceptive pills (OCP) until pregnancy was attempted. Complete response was defined as total absence of malignant cells revealed by D&C after hormone therapy. Time to response was calculated from diagnosis to first benign histopathology result. Time to recurrence was calculated from the time of first benign biopsy to the time of first positive biopsy. For each patient, the total follow- up time, any disease recurrence, residual disease at hysterectomy if performed and the number of viable pregnancies was documented**, **Fig.1.

**Table-I:** Patient treatment, response, recurrence, follow up and outcome of the studied patients

*Patient* *(no.)*	*Age* *(yrs)*	*Treatment*	Primary* *response*	Secondary* *Response*	*Time to response* *(mos)*	*Time to recurrence* *(mos)*	*Follow up* *(mos)*	*Outcome*
1	24	Meg**e**strol Meg**e**strol+GnRHa for recurrence	Yes	---	3	3	20	Mole Hydatidi Form, EP, Hysterectomy, small foci of residual tumor
2	26	Megestrol	No	Yes	6	---	72	Delivery at term, one missed abortion, FOD
3	35	Megestrol	No	No	---	---	---	Hysterectomy, endometrial and concurrent ovarian tumor
4	29	Megestrol	No	Yes	6	18	18	Hysterectomy, endometrial tumor
5	30	Megestrol	No	yes	6	---	48	FOD
6	28	Megestrol Meg**e**strol+GnRH as second- line	No	No	9	---	32	FOD
7	31	Meg**e**strol	No	Yes	6	---	11	FOD
8	32	Meg**e**strol Meg**e**strol+GnRHa for recurrence	No	Yes	6	21	41	Delivery at 36w, hysterectomy, no residual tumor

*The primary and secondary response were defined as positive (yes) if the histopathology result by D&C was benign, or negative (No) if the result revealed carcinoma or hyperplasia with atypical. EP: ectopic pregnancy; FOD: Free of disease

## RESULTS

Between 2002 to 2011, there were 8 patients in the study. The mean age of patients was 30(SD,3.21) years (range 24-35). In six patients, the mean year of infertility was six years (range, 2-10 years). Two patients did not desire to get pregnant and used withdrawal protection method. Three patients had polycystic ovary syndrome. Abnormal uterine bleeding were menorrhagia (two patients), menometrorrhagia (three patients) and oligomenorrhea (three patients). 

All study patients were treated with megestrol acetate alone. Patient treatment, response, recurrence, follow up and outcome are presented in Table-I. Primary response was noted in one patient (12.5%) with atrophic endomertum in the histopathologic result, who was followed by OCP for three months. The follow up D&C showed presence of recurrent tumor. She received the second-line treatment for further three months. After three months with second complete response she was referred to the infertility clinic. Seven patients with no primary response continued hormone treatment for further three months. Of these seven patients, 2 (patients three and 6) had no secondary response. Patient number three requested and underwent hysterectomy with surgical staging. During the surgery, right ovary appeared abnormal. The final hitopathologic result showed an ednometrioid adenocarcinoma FIGO stage IA grade 1 confined to endometrium with concurrent ednometrioid adenocarcinoma of the right ovary. Patient number six received second- line treatment including megestrol acetate (320 mg daily) with GnRHa monthly intramuscular injection. D&C after 3 months did not show any tumor. Of the 8 patients, 7 (87.5%) achieved complete response after hormone therapy, one patient (patient 1) with primary response, 5 patients (patient 2,4,5,7 and 8) with secondary response and one patient (patient 6) with second- line treatment response. The mean time to response was 6.5 months (range 3-9). Of the 7 patients with no primary response 5 (71.5%) achieved secondary response and one with second-line therapy. 

Three of the seven complete responders (patients 1, 4 and 8) developed recurrent disease. The median time to recurrence was 14 months (range 3-21), and the median time to initial response in the patients was 5 months (range, 3-6). One of the 3 patients with recurrent tumor underwent hysterectomy at her own request. The final pathology of the uterus was FIGO stag IA grade 1 endometrioid adenocarcinoma. The remaining 2 (patients 1 and 8) were given the second- line treatment for 3 months. Repeated D&C documented a complete response at 3 months after the second-line treatment. Of the 7 patients with complete remission, 3 (42.8%) conceived. Patient 8 with recurrent tumor conceived by IVF at 35 months from diagnosis and delivered at 36 weeks gestation by cesarean section. She decided to receive a comprehensive surgical staging including hysterectomy after 3 months of delivery.

The histopathologic result revealed no residual tumor. Patient 2 conceived at 15 months of diagnosis by invitro fertilization (IVF) and embro transfer (ET), and delivered at term. Subsequently she conceived spontaneously at 46 months of diagnosis and underwent uterine evacuation for a missed abortion. She insisted on uterus preservation and now is currently free of disease at 6 years after diagnosis. One patient (patient1) had a complete molar pregnancy at 17 months of follow up, and an ectopic pregnancy at 20 months of follow up which was terminated by hysterectomy. The pathologic finding revealed a small foci of superficially grade 1 endometrioid adenocarcinoma of uterus. The remaining patients (patients 5, 6 and 7) are all alive and free of disease. The median follow up of the 7 patients was 34.5 months (range 11-72), with no disease – related death and no major adverse effects with high- dose progestin.

## DISCUSSION

It has been suggested that two different pathogenentic type of endometrial cancer exist according to presence or absence of estrogen as etiologic factor. Type I occurs in younger, perimenopausal women and are estrogenic- dependent tumors than type II. These tumors tend to be stage 1 and low grade.^9^^,^^10^ These patients who wish to preserve fertility, may be treated conservatively with hormone therapy.^11^^,^^12^

High remission rates are seen in well – selected stage I, grade 1 confined to endometrium patients using progestin alone as a conservative treatment.^4^^,^^13^ Many other hormones, such as intrauterine progestins, gonadotropin- releasing hormone analogs (GnRHa), aromatase inhibitors, and selective estrogen receptor modulators have shown their potential in treating as single agent or in addition to progestin therapy.^11^^,^^14^ Therefore we set up a protocol treatment using a potent progestin; megestrol acetate in this study. 

We report on 8 young women, with clinical FIGO stage I grade 1 endometrioid adenocarcinoma confined to endometrium by MRI treated with megstrol alone or second-line treatment, and monitored by repeat D&C at 3 months in a gynecological oncology center. Although an office endometrial biopsy is commonly used to make a definitive diagnosis, this practice may not be as reliable as performance of a D&C in the setting of endometrial malignancy.^15^ We performed D&C as a diagnostic tool, but in one patient a small foci of residual tumor was found in the hysterectomy specimen, despite normal follow- up D&C. Given this result, we suggest to perform an additional diagnostic tool, hysteroscopy examination for the reduction of misdiagnosis.

Many studies regarding progestin treatment of endometrial cancer have comprised case report or small case series in recent years. Their response rates ranging from 62.5 to 89%.^6^^,^^7^^,^^3^^,^^16^^,^^17^

The present study also comprised relatively small case series, but with complete response of 87.5% appears promising. Our result is similar to several studies 78-88%,^5^^,^^18^^,^^19^ but lower than Hyun et al and Niwa et al findings 93-100%,^4^^,^^13^ and higher than the rate reported by some others 62-63%.^2^^,^^17^^,^^20^ The high complete response rate in Hyun et at and Niwa et al studies might be attributable to the more time prolonged treatment period, 3-15 and 6-10 months respectively.

First- line hormone therapy for 3 months failed in 7 of 8 patients, but secondary response was seen in 5 of 7 patients with receiving the protocol treatment for 3 more months. This result is in consistent with Wheeler et al recommendation that a minimum of 6 months treatment is required. There are high affinity receptors for GnRHa in carcinoma of endometrium.^21^ GnRHa has been used adjunctively to progestins as a second- line therapy with successful fertility preserving treatment.^6^ We achieved a complete response for 2 patients by adding GnRHa to megestrol acetate. However, with small sample size in the current study, caution must be applied, and further well designed randomized controlled trials should be done. 

In our series, 3 patients (42.8%) with complete response had recurrence. Two of three patients experienced recurrences within approximately one year after the completion of their treatment. These findings are not consistent with those of Srkalovic et al who found 51% recurrence after 3 years.^22^ In the present study two of three responders with recurrence conceived. Therefore presence of recurrence does not prevent patients to conceive.

The concern is the high potential for synchronous ovarian carcinoma in young patients with endometrial cancer on fertility sparing treatment. We encountered one patient with synchronous ovarian carcinoma among four patients undergoing hysterectomy. In some studies coexisting ovarian malignancy has been reported.^23^^,^^24^ We suggest careful assessment of the ovaries with diagnostic laparoscopy before conservative treatment. Limitation of our study are the small size of patients and performing blind endometrial sampling by D&C.

In conclusion, our findings revealed that the majority of patient with clinical radio-graphic FIGO stage IA G1 endometrioid adenocarcinoma confined to endometrium who underwent fertility sparing treatment had a response. In addition in patients whose tumors recurred, pregnancy was achieved and extrauterine extension was not found, however, close follow- up is required because of the substantial rate of recurrence. We do suggest that therapy for 3 months is insufficient and should not be tried. It is important that the patient is well informed and carefully counseled regarding the risks of conservative treatment.
